# Anxiety Disorder Types From a Metabolomics Perspective: A Mendelian Randomization Analysis Based on 1400 Plasma Metabolites

**DOI:** 10.1002/brb3.71356

**Published:** 2026-03-29

**Authors:** Zhen Xiao, Zedi Wu, Zixin Chen, Ziyue Xu, Qi Zhou, Silan Liu, Muqing Wang, Shujun Hou, Wanling Liu, Zhengyi Li, Kunping Xun, Ning Zhang, Chun Wang

**Affiliations:** ^1^ Nanjing Brain Hospital Affiliated to Nanjing Medical University Nanjing China; ^2^ Taizhou Fifth People's Hospital Taizhou China; ^3^ School of Psychology Nanjing Normal University Nanjing China; ^4^ Cognitive Behavioral Therapy Institute of Nanjing Medical University Nanjing China

**Keywords:** Anxiety, MR, plasma metabolite

## Abstract

**Background:**

Different anxiety types demonstrate overlapping clinical features while retaining distinct characteristics, often leading to diagnostic challenges. Existing diagnostic approaches predominantly rely on symptom‐based criteria, which may result in ambiguity. The identification of biomarkers is essential for elucidating the metabolomic mechanisms underlying anxiety, thereby informing improved diagnostic and therapeutic strategies.

**Methods:**

We conducted a Mendelian randomization (MR) study, utilizing data from genome‐wide association studies (GWAS) on plasma metabolites and three anxiety types. The study meticulously evaluated instrumental variable, as well as heterogeneity, horizontal pleiotropy, and directionality, supplemented by sensitivity analyses.

**Results:**

Initial MR analysis identified common risk metabolites across the three anxiety subtypes. Subsequent sensitivity analyses revealed five specific generalized anxiety disorder (GAD) risk markers, including 3‐hydroxy‐2‐ethylpropionate (OR = 1.248, *P_IVW_* = 0.012) and the glutarate (C5‐DC) to salicylate ratio (OR = 1.314, *P_IVW_* = 0.007). Among social anxiety disorder (SAD) risk markers, 5‐hydroxyhexanoate (OR = 1.005, *P_IVW_* = 0.010) was unique. Potential panic disorder (PD) risk metabolites were identified as arabitol/xylitol (OR = 1.002, *P_IVW_* = 0.011), 2‐hydroxy‐3‐methylvalerate (OR = 1.002, *P_IVW_* = 0.008), and the AMP to phosphate ratio (OR = 1.003, *P_IVW_* = 0.004). Protective factors for GAD included X‐17354 (OR = 0.739, *P_IVW_* = 0.023) and the salicylate to citrate ratio (OR = 0.754, *P_IVW_* = 0.010).

**Conclusion:**

This study highlights potential metabolomic pathways involved in the shared and distinct clinical features of GAD, SAD, and PD. These findings suggest novel biomarkers for developing targeted treatments for anxiety disorders.This study investigates the causal relationship between plasma metabolites and three common types of anxiety disorders using MR analysis. Our findings propose a novel direction for utilizing metabolites as biomarkers in the diagnosis and treatment of anxiety disorders.

## Graphical abstract

This study investigates the causal relationship between plasma metabolites and three common types of anxiety disorders using Mendelian randomization analysis. Our findings propose a novel direction for utilizing metabolites as biomarkers in the diagnosis and treatment of anxiety disorders.

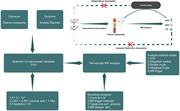

## 1 Introduction

Anxiety disorders represent a significant health problem, as they are among the leading psychiatric causes of the global burden of disease (Huang, Wang et al. 2019; Yang, Fang et al. 2021). Effective prevention and treatment of anxiety disorders are critical to reducing morbidity and disability(Ren et al. 2024). However, successful treatment hinges on accurate diagnosis (Ströhle et al. 2018). GAD, SAD, and panic disorder (PD) share several symptomatic similarities (Craske et al. 2017), which can lead to diagnostic ambiguity and affect treatment decisions (Chen and Lovibond 2020).

Multiple factors, including psychological and genetic components, are believed to contribute to the biological mechanisms underlying anxiety disorders (Shimada‐Sugimoto et al. 2015). Therefore, studying biomarkers beyond symptomatology for diagnostic guidance is warranted. Current research on these three types of anxiety disorders primarily focuses on imaging techniques and neurotransmitter studies (Holzschneider and Mulert 2011; Liu, He et al. 2021; Moraes, Wijaya et al. 2024). However, in the context of diagnostic targets, the ease of obtaining samples and the broad applicability of screening methods are of utmost importance(Loke and Lee 2018).

In recent years, the rapid advancement of plasma metabolomics has offered a new perspective for understanding the biological mechanisms of complex diseases (Lv et al. 2024). By analyzing metabolite changes in plasma, researchers can identify biomarkers related to these diseases, providing a basis for early diagnosis and intervention (Guo, Milburn et al. 2015; Smelik, Zhao et al. 2024). Studies have demonstrated that alterations in metabolites are closely linked to neurochemical processes, inflammatory responses, and individual psychological states, thereby offering substantial data support for further exploration of the biological basis of anxiety subtypes (Shih 2019; Zacharias et al. 2021; Shan, You et al. 2023; Chourpiliadis, Zeng et al. 2024).

Within this field, MR has garnered considerable attention due to its unique advantages. MR utilizes natural genetic variation as an instrumental variable, effectively addressing the confounding and reverse causality issues prevalent in traditional observational studies (Davey Smith and Hemani 2014; Sekula et al. 2016). This approach allows researchers to more reliably infer the causal relationships between plasma metabolites and anxiety disorders, particularly when applied to relatively large independent samples, thus enhancing the reliability of results (Burgess, Foley et al. 2020). Consequently, MR emerges as an ideal tool for exploring the relationships between plasma metabolites and the three anxiety subtypes, including social anxiety, GAD, and PD, presenting new opportunities and challenges for scientific research in the mental health field (Xiao et al. 2022).

## 2 Methods

### 2.1 Data Source

Genetic association data for plasma metabolites were obtained from the GWAS catalog (https://www.ebi.ac.uk/gwas/). Accession numbers for European GWASs: GCST90199621–90201020 (Chen et al. 2023). After strict quality control, 1091 blood metabolites and 309 metabolite ratios were analyzed. In more detail, a total of 8299 participants were recruited from the Canadian Longitudinal Study on Aging (CLSA) cohort. Summary statistics for 3 anxiety subtypes were taken from the OpenGWAS repository (https://gwas.mrcieu.ac.uk/). GWAS of GAD with 200273 sample size (Dataset: finn‐b‐F5_GAD), SAD with 117716 sample size (Dataset: ukb‐d‐20544_1), and PD with 484598 a sample size (Dataset: ebi‐a‐GCST90038651).

### 2.2 Instrumental Variable Selection

**FIGURE 1:**
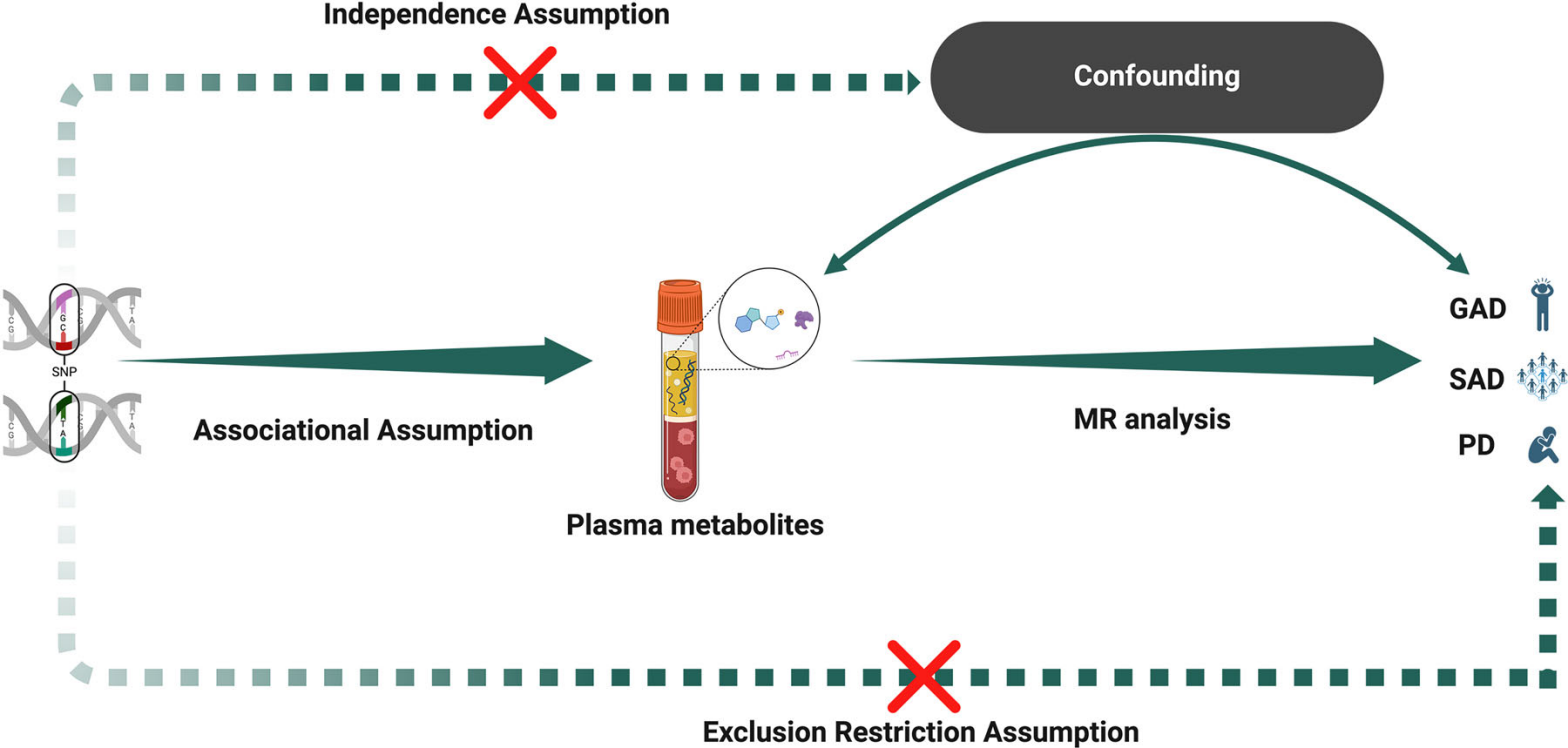
Diagram of MR principles and assumptions. GAD, generalized anxiety disorder; PD, panic disorder; SAD, social anxiety disorder.

MR analysis requires that the IVs satisfy three core assumptions: (1) the IVs are robustly associated with the exposure of interest; (2) the IVs influence the outcome solely through the exposure (no horizontal pleiotropy); and (3) the IVs are independent of any confounders (Scosyrev 2013; Boef, Dekkers et al. 2015) (Figure 1). To ensure that each metabolite had at least one single nucleotide polymorphism (SNP) available as an instrumental variable and to enable comprehensive downstream analyses, an association threshold of *p* < 5 × 10^−^^6^ was applied. Those SNPs with minor allele frequency (MAF) < 0.01 were discarded, then we conducted linkage disequilibrium (LD) pruning (r^2^ threshold = 0.001, window size = 1 Mb) based on the European 1000 Genomes Project reference panel (Cui and Tian 2021). We calculated the strength of genetic instruments using the *F*‐statistic, with *F*‐statistics >10 generally considered to be adequate strength of IVs (Garfield et al. 2023).

### 2.3 MR Analyses

The inverse variance weighted (IVW) model served as our primary analysis method, complemented by four additional models: weighted median, simple mode, weighted mode, and MR‐Egger. This multi‐model approach enhanced the robustness of our causal inference (Burgess, Foley et al. 2020). Heterogeneity across instrumental variables was assessed using Cochran's Q statistic in the IVW and MR‐Egger models (Hemani, G et al. 2018). When significant heterogeneity was detected (Q *p* < 0.05), a random‐effects IVW model was preferentially applied. In addition, leave‐one‐out analyses were conducted to identify potential outlier SNPs contributing to heterogeneity (Bowden et al. 2015). Identified outliers were further examined, and sensitivity analyses were performed to assess the robustness of the causal estimates. To evaluate horizontal pleiotropy, which violates the second instrumental variable assumption by allowing a single genetic variant to influence multiple phenotypes, horizontal pleiotropy was first assessed using the MR‐Egger intercept test. In addition, the MR‐PRESSO (MR Pleiotropy RESidual Sum and Outlier) global test was applied to detect the presence of horizontal pleiotropy and identify potential outlier SNPs. Additionally, the MR‐Steiger directionality test was performed to assess the causal direction between exposures and outcomes. This test compares the variance explained (*R^2^*) by the genetic instruments in the exposure and the outcome. A causal direction from exposure to outcome was considered supported when the instruments explained more variance in the exposure than in the outcome and the corresponding Steiger test *p* value was < 0.05 (Hemani et al. 2017). The MR‐Steiger analysis was applied to exposure–outcome pairs with evidence of a causal association in the primary MR analyses.

In MR studies, sensitivity analyses are crucial for assessing the robustness and reliability of causal relationships. In this study, metabolite results were considered relatively stable and supportive of a potential causal relationship when consistent effect directions were observed across multiple MR methods (e.g., IVW and MR‐Egger), and when sensitivity analyses did not indicate substantial violations of MR assumptions, including absence of significant heterogeneity, horizontal pleiotropy (MR‐Egger intercept and MR‐PRESSO global test), and influence of single SNPs in leave‐one‐out analyses, as well as support for the assumed causal direction in the MR‐Steiger test (Lawlor et al. 2016; Burgess et al. 2017).

## 3 Results

### 3.1 Basic Information of Instrumental Variable

In analyzing the effect of serum metabolites on GAD, the number of generated IVs ranges from 6 to 23 SNPs. In the studies on SAD and PD, the number of IVs ranges from 4 to 23 and 5 to 24 SNPs, respectively. In addition, the minimum *F* statistic of all these IVs was 20.83, suggesting that all IVs were sufficiently effective for the MR analysis (*F* statistic > 10) (Supplementary Material 2).

### 3.2 Causality Between Shared Metabolites and Three Subtypes of Anxiety Disorders

**FIGURE 2:**
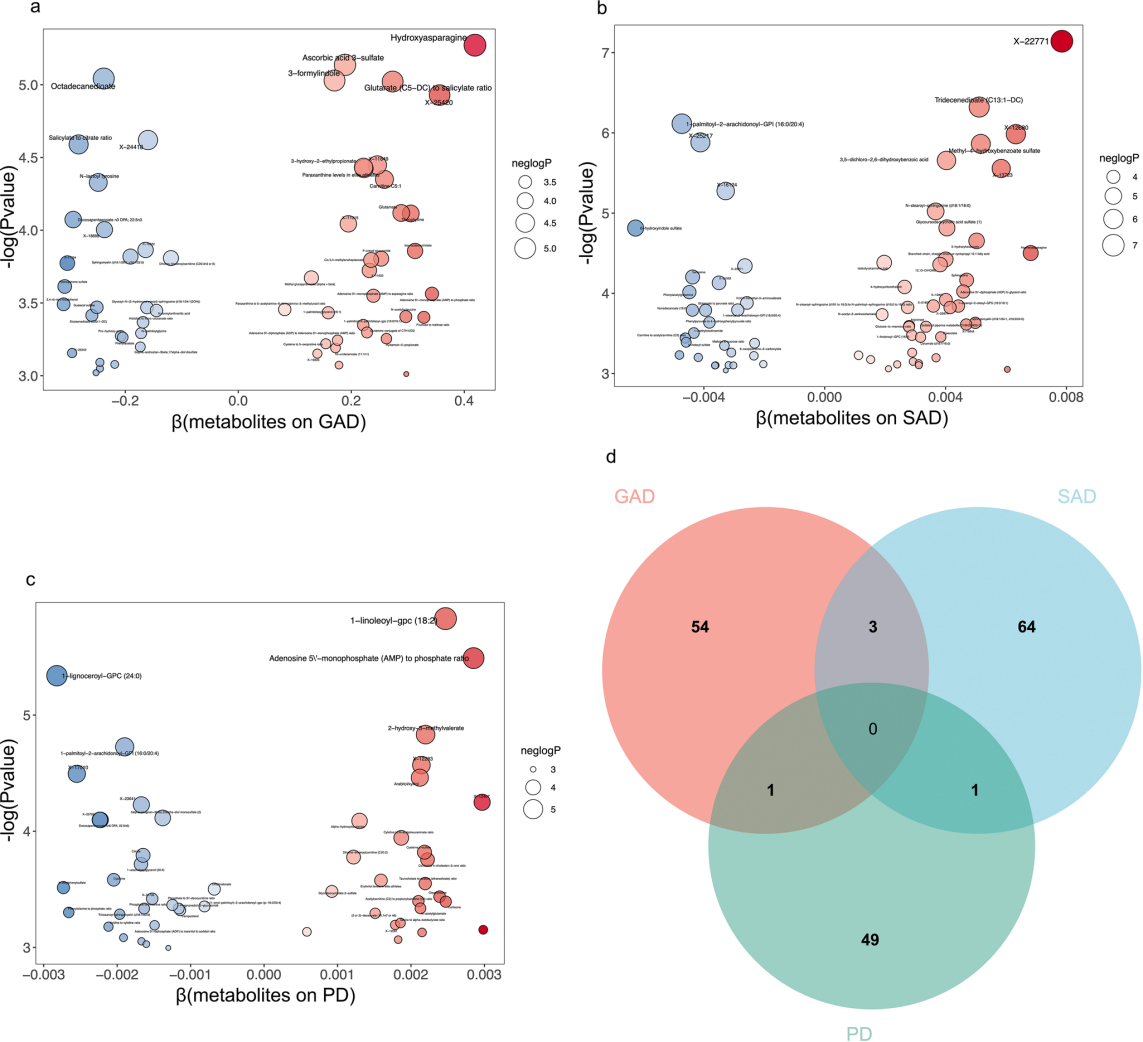
Metabolites with potential causal relationships with three anxiety types. (a) a total of 58 metabolites were identified with potential causal associations with GAD; (b) a total of 68 metabolites were identified in relation to SAD; (c) 51 metabolites were found to influence PD; and (d) the Venn diagram visually represents the overlap of metabolites that exert causal effects on multiple anxiety subtypes, highlighting the shared and unique influences among GAD, SAD, and PD.

The IVW method was employed to validate the causality between 1400 metabolites and anxiety disorders. Figures 2 and S1 illustrate all metabolites identified as having causal relationships with GAD, SAD, and PD, respectively (*P_IVW_* < 0.05). A total of 58 substances were identified with potential causal associations with GAD, of which 32 were classified as risk factors and 26 as protective factors. For SAD, a total of 68 substances were identified (40 associated positively and 28 negatively), while 51 substances influenced PD (25 positively and 26 negatively). Notably, these three anxiety disorders share overlapping metabolic substances. A Venn diagram intuitively illustrates that various metabolites exert causal effects on multiple anxiety subtypes simultaneously (Figure 2d). Both hydroxyasparagine and X‐11315 have the potential to increase the risk of GAD and SAD, while X‐17354 may mitigate this risk. An elevated ratio of adenosine 5'‐monophosphate (AMP) to phosphate is associated with an increased risk of both GAD and PD. 1‐palmitoyl‐2‐arachidonoyl‐GPI is proposed as a protective factor against SAD and PD. Among all analyzed metabolites, no substances were identified that influence all three anxiety subtypes simultaneously.

### 3.3 Potential Diagnostic Targets for Three Anxiety Subtypes

**FIGURE 3:**
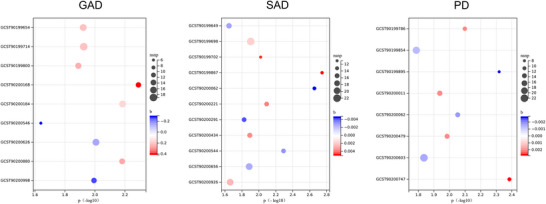
Metabolites that passed all sensitivity analyses. Bubble plots present metabolites that affect each of the three anxiety subtypes separately. b, *β* effect size; p, *p* value; nsnp, number of snp.

In order to obtain potential diagnostic markers usable for the three anxiety subtypes, we conducted a rigorous sensitivity analysis of the previously screened results. The metabolic species considered in this study were deemed relatively stable causal factors affecting anxiety, provided that heterogeneity and horizontal pleiotropy were excluded during the MR analysis. Following this comprehensive sensitivity analysis, nine metabolites exhibited stable causal associations with GAD, with six identified as risk factors and three as protective factors. Additionally, the analysis revealed eleven metabolites exhibiting causal associations with SAD — six with positive and five with negative effects — and eight metabolites influencing PD, four of which were positive. (Figure 3)

**FIGURE 4:**
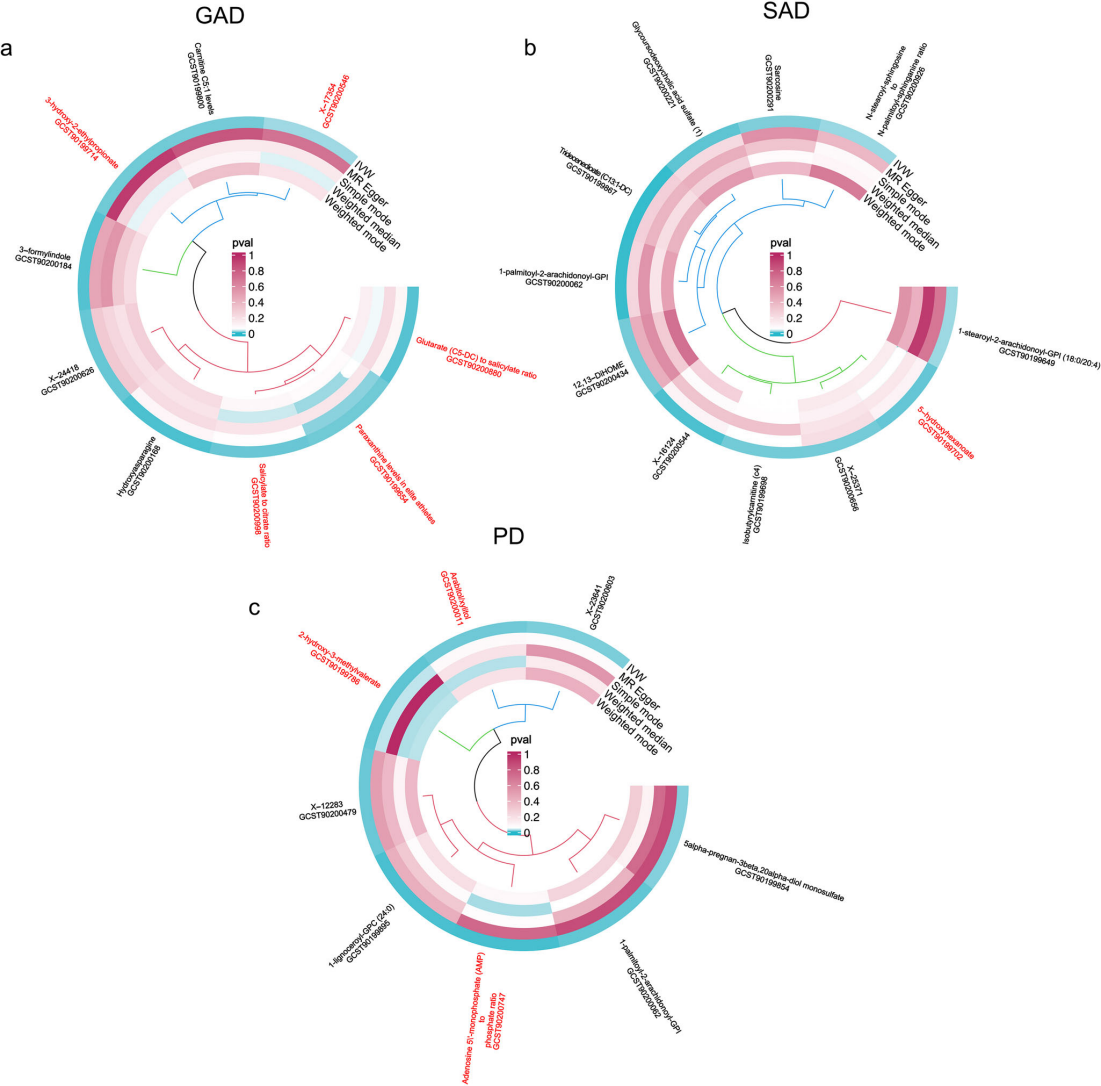
Multiple MR Analysis methods were used to verify the stability of metabolites as causal exposure factors. Metabolites that passed multiple MR Analysis methods are marked in red font.

It is important to emphasize that metabolites demonstrating significant causal effects across multiple MR analysis methods yield results that can be considered more reliable. The analysis identified three metabolites that potentially contribute to an increased risk of GAD: 3‐hydroxy‐2‐ethylpropionate (OR = 1.248, *P_IVW_* = 0.012), the glutarate (C5‐DC) to salicylate ratio (OR = 1.314, *P_IVW_* = 0.007), and paraxanthine levels observed in elite athletes (OR = 1.249, *P_IVW_* = 0.012). Conversely, X‐17354 levels (OR = 0.739, *P_IVW_* = 0.023) and the salicylate to citrate ratio (OR = 0.754, *P_IVW_* = 0.010) exhibited negative correlations with the occurrence of GAD (Figure 4a). Notably, 5‐hydroxyhexanoate (OR = 1.005, *P_IVW_* = 0.010) was the sole metabolite that may increase the risk of SAD (Figure 4b). Among the three metabolites affecting PD, Arabitol/xylitol (OR = 1.002, *P_IVW_* = 0.011), 2‐hydroxy‐3‐methylvalerate (OR = 1.002, *P_IVW_* = 0.008), and the AMP to phosphate ratio (OR = 1.003, *P_IVW_* = 0.004) were all identified as risk factors (Figure 4c).

The sensitivity analysis results for these metabolites are presented in (Table 1) and (Figures S2–S4). Notably, no heterogeneity or horizontal pleiotropy was observed. Furthermore, the Steiger test, which determines the direction of causality, indicated that the effects of the identified metabolites on the three examined subtypes of anxiety were unidirectional.

**TABLE 1:** Sensitivity analysis of causal relationships between 9 metabolites and anxiety types.

Exposure	Outcome	Horizontal pleiotropy			heterogeneity				Steiger	
—	—	—	—	—	MR Egger		IVW		—	—
—	—	Egger intercept	*se*	*P*	*Q*	*P*	*Q*	*P*	Correct causaldirection	Steiger P
X‐17354	GAD	−0.017	0.061	0.788	0.179	0.996	0.262	0.998	True	0.475
3‐hydroxy‐2‐ethylpropionate	GAD	0.029	0.026	0.28	16.9	0.461	18.1	0.446	True	0.192
Salicylate to citrate ratio	GAD	0.028	0.033	0.414	7.57	0.751	8.29	0.762	True	0.369
Paraxanthine levels	GAD	−0.037	0.022	0.113	11.6	0.709	14.4	0.567	True	0.269
Glutarate (C5‐DC) to salicylate ratio	GAD	−0.01	0.028	0.734	5.57	0.936	5.69	0.957	True	0.361
5‐hydroxyhexanoate	SAD	−0.001	0.001	0.423	11.4	0.248	12.3	0.264	True	0.392
Arabitol/xylitol	PD	NA	NA	0.36	3.03	0.998	3.93	0.996	True	0.273
2‐hydroxy‐3‐methylvalerate	PD	NA	NA	0.112	3.27	0.974	6.31	0.852	True	0.159
AMP to phosphate ratio	PD	NA	NA	0.353	5.86	0.754	6.82	0.743	True	0.43

Abbreviations: NA, the statistic is close to zero; *P*, *p* value; *Q*, Cochran^,^s *Q* statistic; *se*, standard error; TRUE, the direction of causality is from exposure to outcome.

## 4 Discussion

In this study, we investigated the causal relationship between metabolites and three types of anxiety disorders: GAD, SAD, and PD, utilizing MR. Previous research has identified causal relationships between 486 plasma metabolites and anxiety disorders (Xiao et al. 2022). Building upon this foundation, the present study enhances the existing knowledge by analyzing 1400 metabolites and examining three common subtypes of anxiety disorders.

Our analysis identified a potential causal link between multiple metabolites and these anxiety subtypes, with several metabolites exhibiting common effects across different anxiety subtypes. In clinical practice, these three anxiety disorders frequently share several characteristics. Although a definitive diagnosis can ultimately be established based on clinical presentation, the overlapping symptoms among these disorders create a risk of misdiagnosis and may influence subsequent treatment decisions. In the first phase, our analysis identified 58 metabolites significantly associated with the risk of GAD, 68 metabolites significantly associated with the risk of SAD, and 51 metabolites significantly associated with the risk of PD. Among these, three metabolites (hydroxyasparagine, X‐11315, and X‐17354) simultaneously influenced both GAD and SAD. Additionally, one metabolite ratio (AMP to phosphate) affected both GAD and PD, while another metabolite (1‐palmitoyl‐2‐arachidonoyl‐GPI) impacted both SAD and PD. Our study did not identify any metabolites that simultaneously influenced all three anxiety subtypes. These overlapping influences suggest potential shared mechanisms underlying different anxiety subtypes, highlighting the complex interplay between metabolites and anxiety disorders.

In the aforementioned overlapping metabolites, X‐11315 and X‐17354 have been insufficiently studied in terms of their functions, necessitating further research. The AMP to phosphate ratio is a critical biochemical indicator that elucidates cellular energy state and metabolic conditions. Previous studies have demonstrated that this indicator is strongly associated with metabolic diseases (Hardie 2003), cardiovascular diseases (Turner et al. 2024), and neurodegenerative diseases (Takaine, Imamura et al. 2022). Furthermore, it may function as a diagnostic marker for assessing cellular energy status and metabolic health, indicating its potential as a diagnostic target for mental disorders. In our study, this indicator was implicated in both GAD and PD. Notably, the cardiac discomfort frequently reported by these patients could potentially be associated with this indicator. Future research should further investigate the association between this indicator and specific clinical symptoms, particularly focusing on its role in anxiety‐related cardiac manifestations.

The final metabolite identified in our study that is implicated in multiple anxiety subtypes is 1‐palmitoyl‐2‐arachidonoyl‐GPI. This significant biological molecule exhibits a pivotal role in cell membrane function, signal transduction, and protein localization (Kinoshita 2020). Although direct evidence linking it to specific diseases is currently lacking, alterations in its levels or function may be associated with various pathological states. Given its potential importance, further research is necessary to elucidate specific connections between 1‐palmitoyl‐2‐arachidonoyl‐GPI and anxiety disorders, as well as other related mental health conditions.

Since one of the primary objectives of the study was to identify potential biomarkers capable of effectively differentiating among these three distinct anxiety disorders, we performed an analysis of the existing results in the second phase. Through a rigorous sensitivity analysis, we identified more robust findings, which revealed a total of five metabolites associated with the risk of GAD, one metabolite associated with the risk of SAD, and three metabolites associated with the risk of PD. These relatively stable findings may serve as potential biomarkers for the diagnosis and differentiation of the three anxiety subtypes beyond their symptomatology.

Of the five potential GAD biomarkers identified, X‐17354 and 3‐hydroxy‐2‐ethylpropionate remain understudied. However, other metabolites have been previously reported in the field of psychiatric disorders.

Paraxanthine, like caffeine, is classified as a psychoactive central nervous system stimulant(Orrú et al. 2013). It may enhance the release of glutamate and dopamine by stimulating nitric oxide signaling, and alterations in these neurotransmitters are closely associated with various mental disorders (Howes et al. 2015). Studies have demonstrated that salicylates can induce anxiety‐like behaviors, particularly in young individuals, possibly due to alterations in brain activity in thalamic and cortical regions involved in emotional processing and sensory perception (Chen, Wang et al. 2024). Although our study does not establish a causal relationship between salicylate levels and various anxiety disorders, we found significant associations between the glutarate (C5‐DC) to salicylate ratio and the salicylate to citrate ratio with the risk of GAD.

5‐hydroxyhexanoate, a metabolite identified in this study as potentially increasing the risk of SAD, lacks direct evidence for a clear relationship with psychiatric diseases in prior studies. However, it may indirectly influence nervous system function by impacting energy metabolism in the brain, membrane function, or through other unknown mechanisms (Rae, Baur et al. 2024). Further research is necessary to elucidate its precise role in psychiatric disorders.

Among the three metabolites associated with PD, 2‐hydroxy‐3‐methylvalerate may indirectly influence brain function by altering the balance of neurotransmitters. Our finding that the AMP to phosphate ratio increases the risk of PD aligns with previous studies indicating that serum phosphate levels are inversely associated with anxiety and depressive symptoms (Aalbers et al. 1990), and that hypophosphatemia may be linked to PD (Roestel, Hoeping et al. 2004). Lastly, xylitol is associated with gut microbiota, and recent research on the gut‐brain axis has demonstrated that gut microbiota can influence anxiety disorders(Xiang, Ye et al. 2021; Butler et al. 2023).

A prior study identified 1‐linoleoylglycerophosphoethanolamine as a potential risk factor for anxiety disorders(Xiao et al. 2022). However, our investigation failed to establish a causal relationship between this metabolite and the three specific anxiety subtypes under examination. This apparent incongruity may be attributed to methodological differences, particularly in the selection of outcome sample databases. Notably, the aforementioned study utilized outcomes from patients diagnosed with GADs without delineating specific subtypes, whereas our study focused on distinct anxiety subtypes. This discrepancy not only highlights the heterogeneity within anxiety disorders but also underscores the limitations of traditional symptom‐based diagnostic criteria for psychiatric disorders. The divergent findings between these studies emphasize the complexity of anxiety disorders and suggest that risk factors may vary across subtypes.

At present, the diagnosis of many psychiatric disorders, including anxiety disorders, predominantly relies on symptom‐based criteria, leading to significant diagnostic ambiguity. This ambiguity substantially impacts treatment approaches and prognostic outcomes, highlighting the need for more objective diagnostic methods. The identification of specific metabolites as auxiliary diagnostic markers presents a promising solution, with the potential to enhance diagnostic accuracy and precision. Moreover, these metabolic markers may serve dual purposes: not only as diagnostic tools but also as potential therapeutic targets. This dual functionality could inform future research directions for anti‐anxiety medications, potentially leading to more effective treatments. Ultimately, such advancements may mitigate the economic burden associated with anxiety disorders by improving treatment efficacy and reducing misdiagnoses.

Future studies should build upon the identified metabolites, and additional experiments need to be designed to validate the findings in animal models. This will facilitate the investigation of the underlying pathological mechanisms of these metabolites in the onset and progression of various anxiety disorders. Furthermore, clinical plasma samples from these three patient groups could be collected for cross‐sectional studies to determine the real‐world significance of the findings from this study.

In conclusion, this study has revealed potential causal relationships between various metabolites and GAD, SAD, and PD through MR analysis. These findings deepen our understanding of the pathogenesis of anxiety disorders and provide new avenues for developing future diagnostic and therapeutic strategies. Nonetheless, the results of this study should be regarded as preliminary and hypothesis‐generating, necessitating further validation in subsequent research.

Limitation: While this study provides valuable theoretical causal inferences based on existing GWAS databases, it is important to acknowledge its limitations. Firstly, the results have not been empirically validated in real‐world settings, which is crucial for confirming their clinical relevance. Secondly, the exclusive use of European samples in the databases introduces a significant constraint. This lack of ethnic diversity may limit the generalizability of the results to other populations, potentially overlooking genetic variations and environmental factors specific to non‐European groups. Future research should address these limitations by incorporating diverse population samples and conducting real‐world validation studies to enhance the robustness and global applicability of these findings.

## 5 Conclusion

This MR study elucidated a potential metabolomic mechanism underlying the common clinical features of GAD, SAD, and PD. Furthermore, nine specific plasma metabolites demonstrating potential causal relationships with the three anxiety subtypes were identified. These findings suggest a new direction for using metabolites as biomarkers in the diagnosis and treatment of anxiety disorders.

## Author Contributions

**Zhen Xiao**: writing – original draft, visualization, investigation, data curation, conceptualization. **Zedi Wu**: data curation, data analysis, revision of article. **Zixin Chen**: writing – original draft, data curation. **Ziyue Xu**: data curation. **Qi Zhou**: data curation. **Silan Liu**: data curation. **Muqing Wang**: data curation. **Shujun Hou**: data curation. **Wanling Liu**: data curation. **Zhengyi Li**: data curation. **Kunping Xun**: data curation. **Ning Zhang**: revision of article. **Chun Wang**: writing – review and editing, Supervision.

## Funding

This study was supported by the National Natural Science Foundation of China (81971289 and 81571344).

## Ethics Statement

All summary‐level datasets used in our study were publicly available, with ethical approval and informed consent already obtained from the respective institutions (the research ethics boards of the Jewish General Hospital, protocol number 2021–2762).

## Conflicts of Interest

The authors declare no conflict of interest.

## AI Usage Declaration

Artificial intelligence tools (ChatGPT) were used only for language editing and improving readability. The authors take full responsibility for the content of the manuscript.

## Supporting information




**Supplementary Figures**: brb371356‐sup‐0001‐figureS1‐S4.docx

**Supplementary Material**: brb371356‐sup‐0002‐SuppMat.xlsx

## Data Availability

For inquiries regarding access to the datasets, kindly contact the corresponding author, Chun Wang, at chun_wang@njmu.edu.cn
