# Supplementary material for: Anxiety Disorder Types From a Metabolomics Perspective: A Mendelian Randomization Analysis Based on 1400 Plasma Metabolites
Source: Brain Behav. 2026 Mar 29;16(4):e71356. doi: 10.1002/brb3.71356 (PMC13112010; doi:10.1002/brb3.71356)
Supplement: Supplementary file 1 — Supplementary Figures: brb371356‐sup‐0001‐figureS1‐S4.docx [file BRB3-16-e71356-s001.docx]

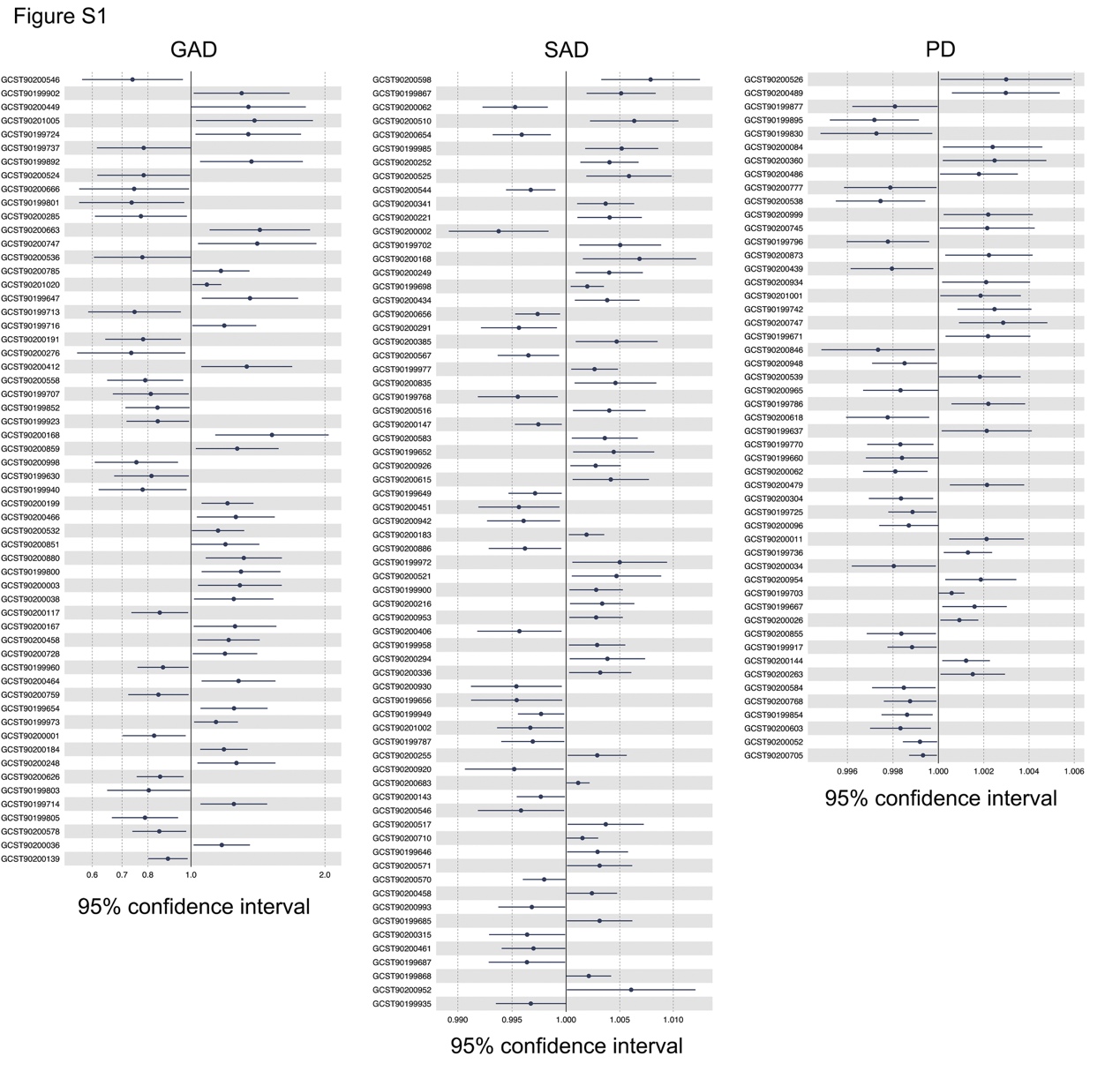


Figure S1. The forest plot presents all metabolites that presented a potential causal relationship for the three anxiety subtypes in the first stage analysis.


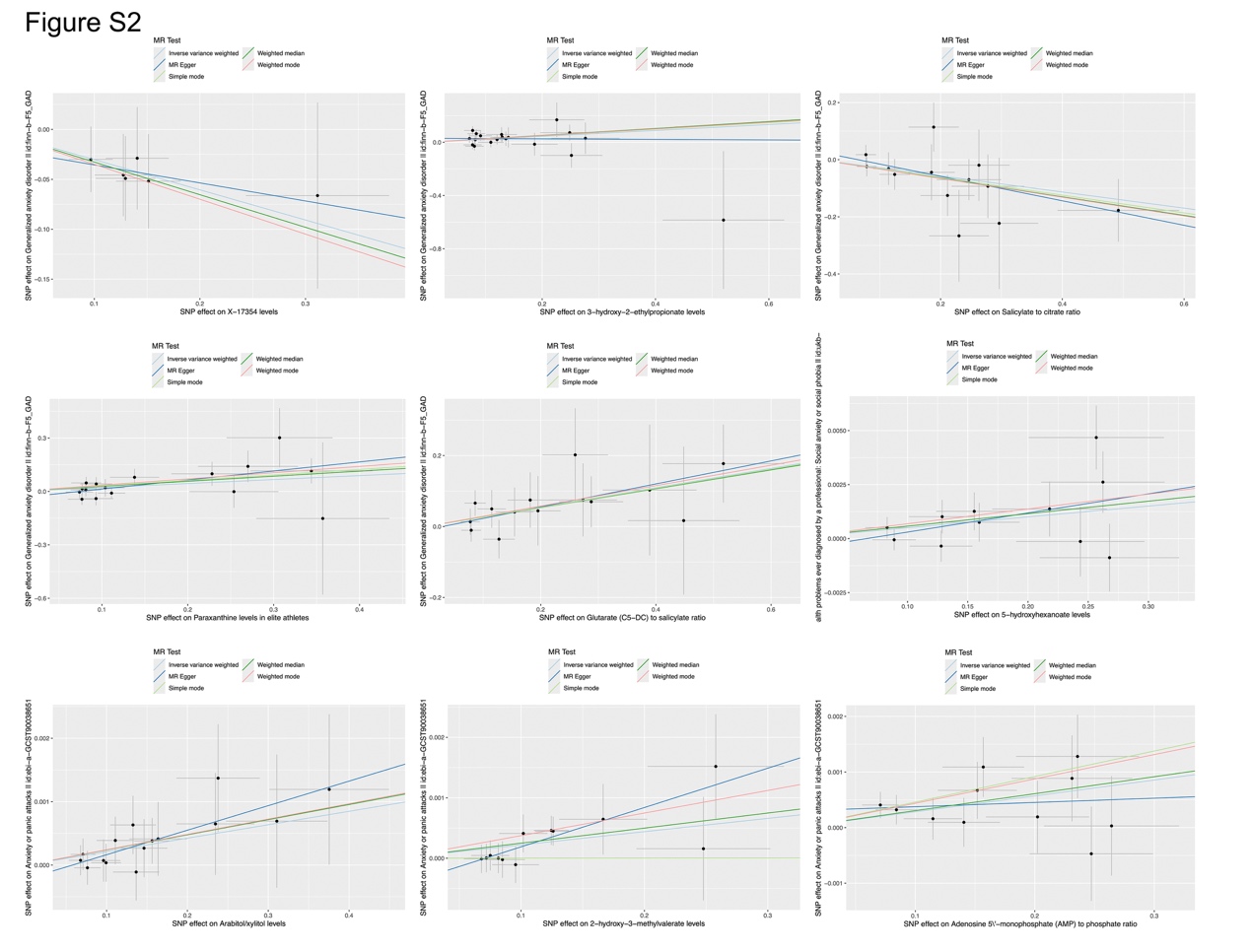


Figure S2. Scatter plots for MR analyses of the causal effect of metabolites on anxiety. The slope of each line corresponds to the estimated MR effect per method.


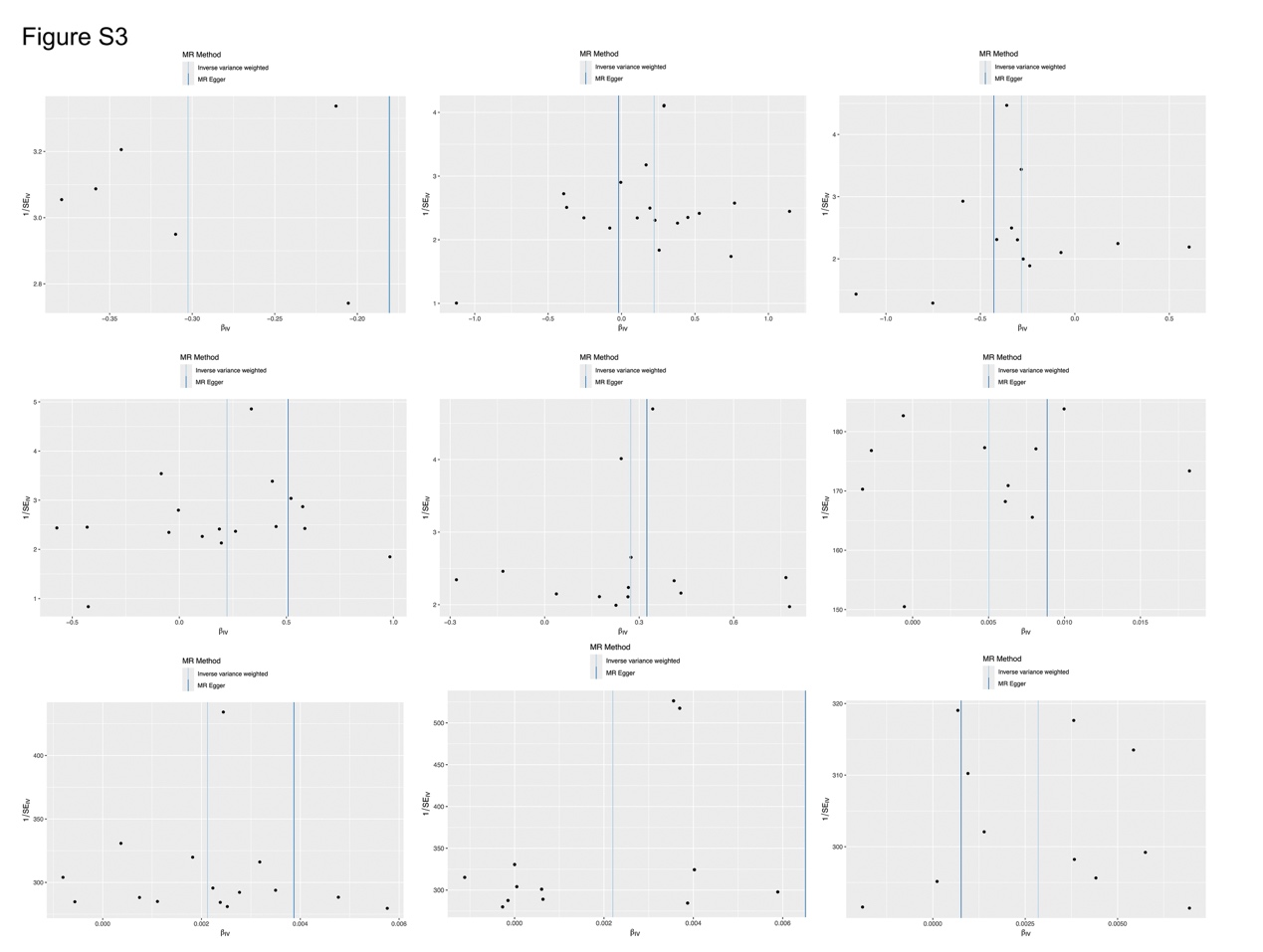


Figure S3. The funnel plot visually presents the heterogeneity in the MR Analysis


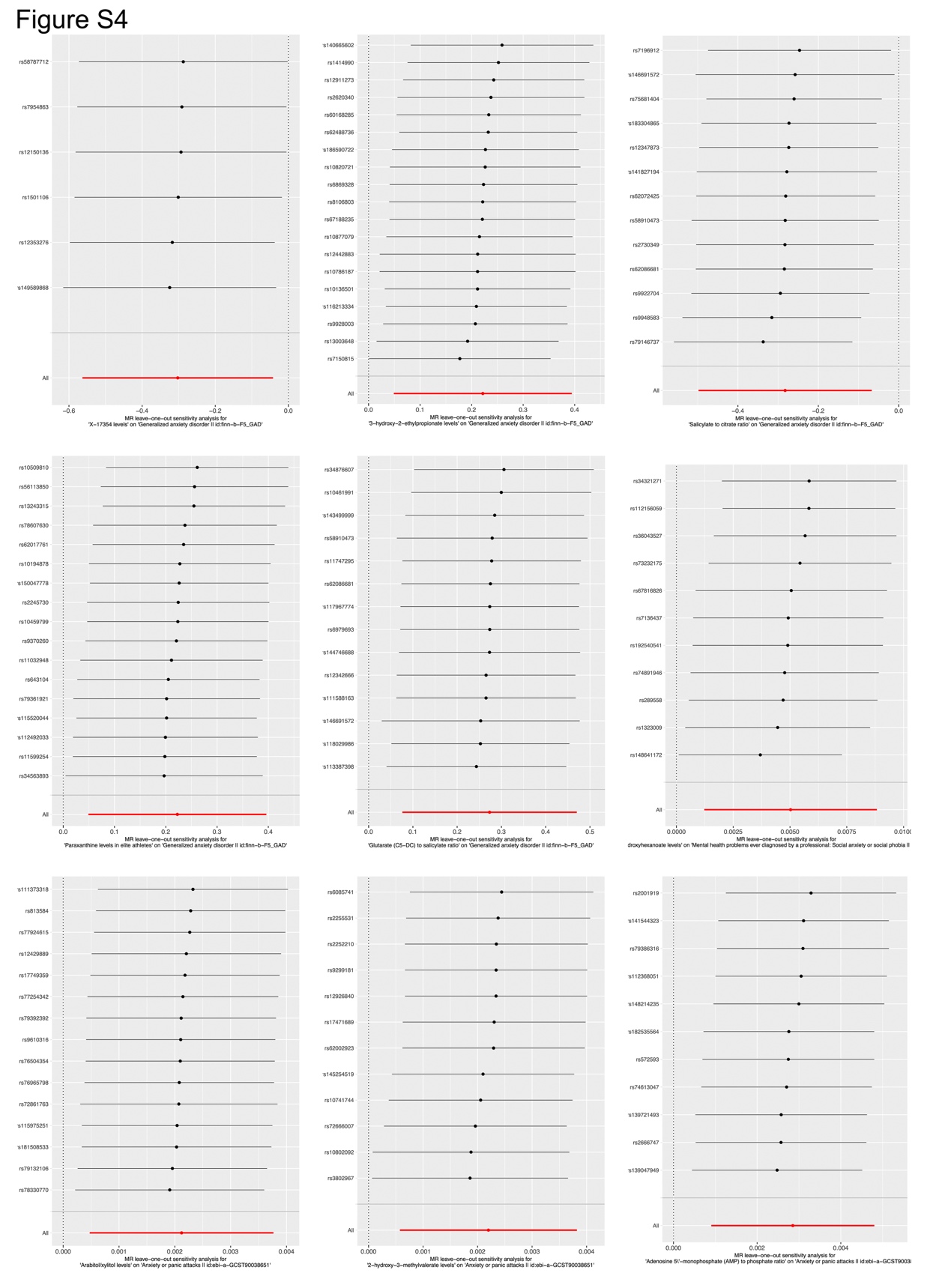


Figure S4. Leave-One-Out Analysis of Causal Effects of Metabolites on Anxiety. Each black point in the plot represents the estimate of the causal effect of metabolite levels on anxiety, derived from the IVW method, while excluding a specific variant from the analysis. Red Point: The red point indicates the IVW estimate calculated using all SNPs, providing a comprehensive view of the overall causal effect.
